# Current Classification of Canine Muscular Dystrophies and Identification of New Variants

**DOI:** 10.3390/genes14081557

**Published:** 2023-07-29

**Authors:** G. Diane Shelton, Katie M. Minor, Steven G. Friedenberg, Jonah N. Cullen, Ling T. Guo, James R. Mickelson

**Affiliations:** 1 Department of Pathology, School of Medicine, University of California San Diego, La Jolla, CA 92093, USA; liguo@ucsd.edu; 2 Department of Veterinary and Biomedical Sciences, College of Veterinary Medicine, University of Minnesota, Saint Paul, MN 55108, USA; minork@umn.edu (K.M.M.); micke001@umn.edu (J.R.M.); 3 Department of Veterinary Clinical Sciences, College of Veterinary Medicine, University of Minnesota, Saint Paul, MN 55108, USA; fried255@umn.edu (S.G.F.); cull0084@umn.edu (J.N.C.)

**Keywords:** dog, muscle, myopathy, whole genome sequencing, animal model

## Abstract

The spectrum of canine muscular dystrophies has rapidly grown with the recent identification of several more affected breeds and associated mutations. Defects include those in genes and protein products associated with the sarcolemma (dystrophin deficient X-linked muscular dystrophy and sarcoglycan-deficient limb–girdle muscular dystrophy) and with the extracellular matrix (collagen 6, laminin α2, and α-dystroglycan-deficient congenital muscular dystrophies). With the increasing application of whole genome sequencing and whole exome sequencing, the clinical and pathological spectra associated with specific neuromuscular genetic defects are constantly evolving. In this report, we provide a brief overview of the current status of gene defects reported in canine muscular dystrophies. We also report the causative mutations for novel forms of X-linked muscular dystrophy in Brittany spaniels and in a French bulldog.

## 1. Introduction

The expansion of molecular testing in people and animals has led to the identification of many gene defects and protein products associated with neuromuscular disorders. With the increasing application of whole genome sequencing (WGS) and whole exome sequencing, the clinical and pathological spectra associated with established neuromuscular genetic defects are constantly evolving, while new variants, ranging from uncertain to likely functional significance, continue to be identified. In these latter cases, muscle pathology plays a key role in determining if a new variant is likely to be pathogenic. Genes encoding proteins throughout muscle fibers have now been shown to be responsible for neuromuscular disorders. These include proteins located in the extracellular matrix, the plasma membrane, the cytoskeleton, the Golgi, the internal membrane systems, nuclei, myofibrils, and the neuromuscular junction.

In people, the discovery in 1987 of the gene encoding dystrophin (*DMD*), responsible for X-linked Duchenne and Becker muscular dystrophy, paved the way for identifying and characterizing the role of many other sarcolemmal and extracellular matrix proteins, such as the sarcoglycans and laminin α2, and their own roles in other forms of muscular dystrophies [1]. Defects in other membrane proteins including dysferlin, caveolin 3, telethonin, as well as the enzyme calpain 3 also result in forms of limb–girdle and congenital muscular dystrophies [2]. Mutations in nuclear membrane proteins such as emerin, lamin A/C and nesprins are responsible for Emery–Dreifuss forms of muscular dystrophy [3]. Further, a growing number of genes and their protein products involved in post-translational glycosylation pathways, in particular of α-dystroglycan, are responsible for human neuromuscular disorders and various forms of congenital muscular dystrophy [4].

Muscular dystrophies in people are currently classified as the Duchenne and Becker muscular dystrophies associated with mutations in the *DMD* gene, the limb–girdle muscular dystrophies with either autosomal dominant or autosomal recessive modes of inheritance and associated with mutations in the sarcoglycans and other genes, the congenital muscular dystrophies associated with mutations in collagen 6, laminin α2 and others, Emery–Dreifuss muscular dystrophy, and facioscapulohumeral, myotonic and oculopharyngeal muscular dystrophies [5].

The identification and molecular characterization of muscular dystrophies is similarly growing in veterinary medicine (Table 1, [6,7,8,9,10,11,12,13,14,15,16,17,18,19,20,21,22,23,24,25,26,27,28,29,30,31]). The first mutation in the *DMD* gene encoding dystrophin and causing X-linked MD was identified in 1992 by Sharp et al. in the Golden Retriever [6]. Since that time, numerous *DMD* variants including deletions, insertions, nonsense or stop gain variants, and larger structural variants including inversions and tandem duplications have been described in several breeds of dogs (Table 1, [7,8,9,10,11,12,13,14,15,16,17,18,19,20,21,22,23,24,25,26,27,28,29]). The spectrum of muscular dystrophies in dogs has also expanded in recent years to include forms of limb–girdle muscular dystrophy associated with variants in sarcoglycan genes (*SGCD* and *SGCA*) and congenital muscular dystrophies with variants identified in the *COL6*, *LAMA2*, and *LARGE1* genes [22,23,24,25,26,27,28,29]. The list is sure to grow, as molecular techniques are increasingly becoming available at a reasonable cost.

In addition to rapidly identifying new pathogenic gene candidates, next-generation sequencing in dogs is also widening the spectrum of clinical phenotypes associated with well-established disease-causing genes. This in turn is increasingly challenging the traditional classification of neuromuscular diseases based primarily on clinical features. There is often considerable clinical and pathological overlap between disorders, and it can now be the case that multiple variants can occur in the same gene in the same breed and result in variable clinical presentations. As such, a strong case can now be made that congenital neuromuscular diseases should be classified based on the protein or gene defect, for example, dystrophinopathy or sarcoglycanopathy, and according to the pathogenic mechanisms.

Dystrophin-deficient MD is currently the most common form of MD in dogs, although forms of LGMD [22,23] and CMD [24,25,26,27,28,29] have recently been recognized. It is difficult, if not impossible, to determine the specific form of canine MD (i.e., XLMD, LGMD or CMD, among others) based on clinical signs alone. A careful clinical assessment including measurement of CK activity and histological evaluation of muscle biopsies can point the clinician in the right direction for identification of a causative protein or gene defect. Here, we utilize this combined pathological approach coupled with WGS to identify and report the causative mutations for novel forms of X-linked MD in Brittany spaniels and in a French bulldog.

## 2. Materials and Methods

### 2.1. Animals

DNA was evaluated from two young, previously reported male Brittany spaniel dogs, one from the USA and one from Japan, with dystrophin-deficient muscular dystrophy based on clinical presentation, markedly elevated creatine kinase (CK) activity, histopathology and immunofluorescent staining of dystrophy-associated proteins [30]. An additional dog, a 3-month-old male French bulldog, was evaluated for dysphagia, dyspnea and macroglossia, with muscle biopsies submitted. Genomic DNA from all three dogs was isolated from archived frozen diagnostic muscle biopsy specimens using the Qiagen DNEasy kit according to package instructions.

### 2.2. Light Microscopy and Immunofluorescent Staining

Histopathological and immunofluorescent characterizations of muscle biopsies from both Brittany spaniels were previously described [30]. Unfixed, chilled and formalin-fixed diagnostic muscle biopsy specimens were collected post-mortem from the biceps femoris, diaphragm and tongue muscles of the French bulldog and shipped by an overnight express service under refrigeration to the Comparative Neuromuscular Laboratory, University of California San Diego. Upon receipt, the unfixed muscle specimens were snap frozen in isopentane, pre-cooled in liquid nitrogen, and stored at −80 °C until further processed. Cryosections were evaluated using a standard panel of histochemical stains and reactions [31]. Additional cryosections were cut and stained for indirect immunofluorescence as previously described [32], using monoclonal antibodies against the rod (1:100, NCL-DYS1) and carboxy-terminus (1:100, NCL-DYS2) of dystrophin, utrophin (1:20, NCL-DRP2), and developmental myosin heavy chain for regenerating fibers (1:20, NCL-dMHC), all from Novocastra Laboratories, Newcastle, UK; a monoclonal antibody against caveolin 3 (1:100, Santa Cruz Biotechnology Inc., Santa Cruz, CA, USA), and polyclonal antibodies against laminin α2 (1:200), α-sarcoglycan (1:200), and collagen VI (direct apply, monoclonal antibody 3G7), all gifts of Professor Eva Engvall [33,34].

### 2.3. Whole Genome Sequencing and Variant Analysis

A PCR-free library was prepared from all three cases, and 150 bp paired-end reads were generated on an Illumina HiSeq 4000 sequencer by GeneWiz (South Plainfield, NJ, USA). For one Brittany spaniel, 767.6 million paired-end reads were generated, corresponding to a mean 45.1 fold genome-wide coverage; for the second Brittany spaniel, 382.5 million paired-end reads were generated, corresponding to a mean 22.5 fold genome-wide coverage; and for the French bulldog, 314.1 million paired-end reads were generated, corresponding to a mean 18.4 fold genome-wide coverage. Reads were mapped against the dog reference genome assembly (CanFam4) and processed using the OnlyWAG pipeline as described [35]. Raw sequence reads are available in NCBI’s Short Read Archive at BioProject PRJNA982829. 

WGS variants from the affected dogs were compared to those of control genomes from an internal WGS database developed at the University of Minnesota and containing 671 dogs of 63 diverse breeds (including 3 additional Brittany spaniels and 22 French bulldogs from unrelated projects). The WGS data from these 671 dogs were processed using the same bioinformatics pipeline referenced above, and included dogs from the following breeds: Golden Retriever (n = 46), Yorkshire Terrier (n = 42), Miniature Schnauzer (n = 38), Boxer (n = 37), English Bulldog (n = 34), Standard Poodle (n = 28), German Shepherd (n = 27), Portuguese Water Dog (n = 25), Great Dane (n = 25), Cavalier King Charles Spaniel (n = 23), Labrador Retriever (n = 22), French Bulldog (n = 21), Irish Wolfhound (n = 20), Bull Mastiff (n = 19), Rottweiler (n = 17), Siberian Husky (n = 16), Whippet (n = 15), Newfoundland (n = 15), Dachshund (n = 15), Pomeranian (n = 13), Scottish Deerhound (n = 10), Miniature Poodle (n = 10), Bouvier (n = 10), Border Collie (n = 10), and 133 dogs of 39 additional breeds, each with less than 10 dogs per breed. Variants unique to the affected dogs that were within or in close proximity to coding exons were prioritized as high (frame shift, loss or gain of stop or start codon, affecting a splice junction), moderate (missense), or low (synonymous, near splice junction) for further evaluation. A list of the unique coding variants of each dog is provided as Table S1.

### 2.4. Sanger Sequencing

Sanger sequencing of a PCR amplicon was employed to genotype a relative of Brittany spaniel case 1 for an identified *DMD* point mutation. This PCR utilized standard conditions, with primers DMD exon 55F (CCCTCTGCCTCTTTCCTTCT) and DMD exon 55R (AGCAACAACCCATACCCTTG) producing a 476 bp product.

Sanger sequencing was also employed on two PCR amplicons to confirm the boundaries of a hypothesized insertion in the *DMD* gene in Brittany spaniel case 2. PCR utilized standard conditions, with primers for the first amplicon being DMD intron 20F (TGACCCACCTCTCTCATTCTG) and RSPRY1 exon 3R (TTCTGCCATTTCGTGCAA), producing a 701 bp product, and for the second amplicon being RSPRY1 3′UTR F (GTACCTCAGCAGCTGCCTTT) and DMD intron 20R (AGCCCCCTAAACAGGAAGAA), producing a 430 bp product.

Additionally, Sanger sequencing was employed to genotype an archived French bulldog with a dystrophic phenotype for the DMD 1 bp insertion (p.F1125*) variant identified in the French bulldog case presented in this manuscript. PCR utilized standard conditions, with primers DMD exon 25F (AGCCTTCAAGGAGGTCGTTT) and DMD exon 25R (CAATTTTTGCAAGGTTGAGACA) producing a 595 bp product.

## 3. Results

### 3.1. Animals

The history, clinical presentation, and laboratory findings in both young male Brittany spaniels were previously reported [30]. Dystrophin-deficient MD was confirmed in both cases by the undetectable labeling of the rod and carboxy terminus of dystrophin using immunofluorescent staining. A 3-month-old male intact French bulldog was evaluated for dysphagia and dyspnea from severe macroglossia, low head carriage and tetraparesis. Progressive muscle contracture resulted in an inability to open the jaw or stand. The CK activity was reported as elevated. The dog was euthanized, and muscles collected for further evaluation.

### 3.2. Light Microscopy and Immunofluorescent Staining

**Figure 1:**
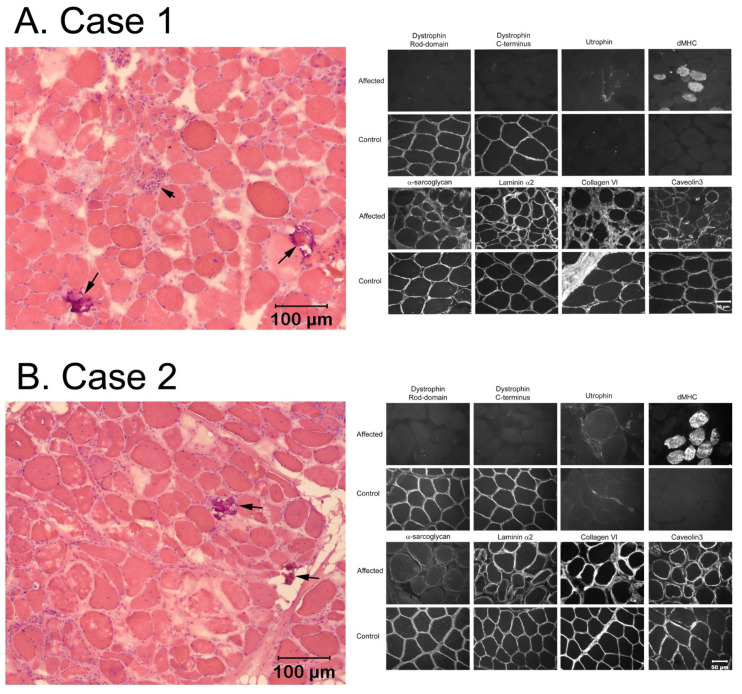
Histopathology and immunofluorescent staining of muscle cryosections from two Brittany spaniels with dystrophin-deficient muscular dystrophy. (**A**) is Case 1, a 6-month-old intact male Brittany from the USA, and (**B**) is Case 2, a 7-month-old intact male Brittany from Japan. In (**A**), H&E stained cryosections (**left**) show variability in myofiber size, myonecrosis (arrowhead) and calcific deposits (arrows) consistent with a degenerative myopathy. Immunofluorescence staining for dystrophy-associated proteins (**right**) shows absent or markedly decreased staining for the rod and carboxy terminus of dystrophin, upregulation of utrophin, and clusters of regenerating fibers using the dMHC antibody for regenerating fibers. In (**B**), H&E stained cryosections (**left**) show variability in myofiber size and calcific deposits (arrows). Immunofluorescent staining for dystrophy-associated proteins showed a staining pattern similar to that of Case 1, with undetectable staining for the rod and carboxy terminus of dystrophin, upregulation of utrophin, and clusters of regenerating fibers (dMHC antibody). Staining for other dystrophy-associated proteins, including those for laminin α2, collagen 6, and caveolin 3, was similar to control in both cases.

**Figure 2:**
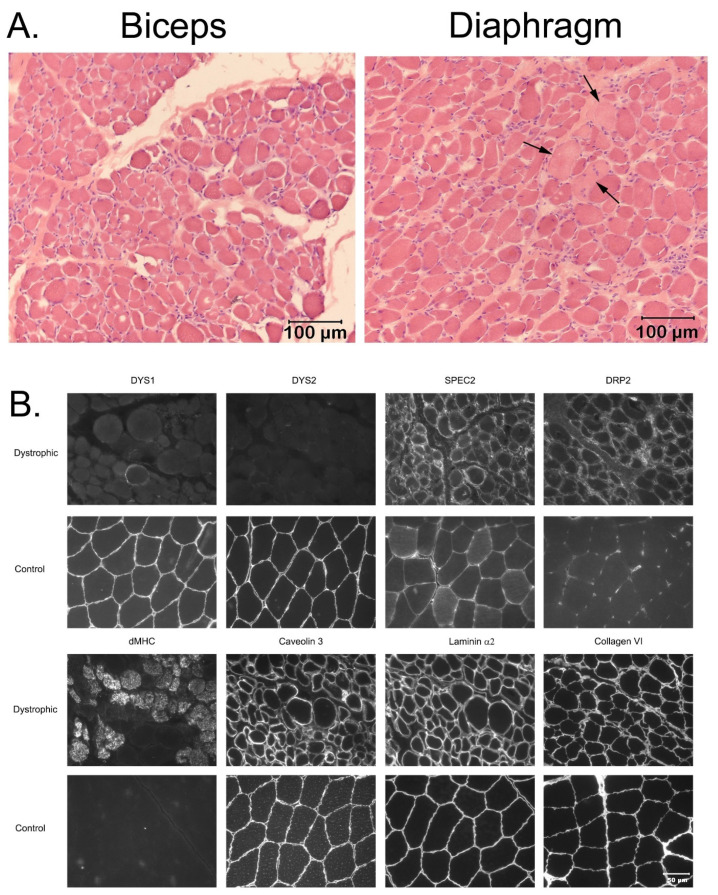
Histology and Immunofluorescent staining of muscle cryosections from a French Bulldog. (**A**) Cryosections from the dystrophic French bulldog biceps femoris muscle and diaphragm showing degenerative and regenerative changes consistent with a form of muscular dystrophy. Variability in myofiber size was present in both muscles, with scattered and pale staining necrotic fibers (arrows) present in the diaphragm. H&E stain, bar = 100 µm for both images. (**B**) Immunofluorescent staining of muscle cryosections from the dystrophic French bulldog and an archived non-age matched control muscle using antibodies against dystrophy-associated proteins. Staining was absent for the rod (DYS1) and carboxy-terminus (DYS2) of dystrophin, with upregulation of utrophin (DRP2) consistent with a diagnosis of a dystrophin-deficient muscular dystrophy. Regenerating muscle fibers are highlighted with the antibody against developmental myosin heavy chain (dMCH). Results of staining for caveolin 3, laminin α2 and collagen VI are similar to those for control muscle. Bar in the lower right corner = 50 µm for all images.

A degenerative (pale staining myofibers consistent with myonecrosis) and regenerative (regenerating fibers confirmed with the antibody against dMHC) myopathy was identified in the muscles of all three cases consistent with a dystrophic phenotype (H&E stained muscle cryosections of the Brittany spaniel Cases 1 and 2 are shown in Figure 1A and reference [30], and H&E stained cryosections from the biceps muscle and diaphragm of the French bulldog are shown in Figure 2A). Scattered calcific deposits were evident in Cases 1 and 2 but not in the French Bulldog. Compared to archived control muscle, immunofluorescent staining was absent for the rod- and carboxy-terminus of dystrophin in all three cases (Figure 1B and Figure 2B), with upregulation of utrophin consistent with a diagnosis of a dystrophin-deficient muscular dystrophy.

### 3.3. Two Novel DMD Variants Identified in Dystrophin-Deficient Brittany Spaniels

**Figure 3:**
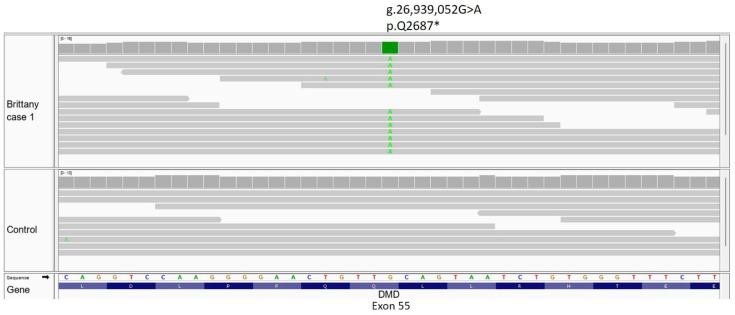
IGV view of a *DMD* point mutation in exon 55 of Brittany spaniel Case 1. The green base identified in all reads covering this base in the case is the replacement of a G at genomic position 26,939,052 with an A, resulting in a p.Q2687* mutation in the DMD protein.

Case 1 (USA Brittany spaniel) reported in 2022 [30]. Examination of this variant call format (VCF) for unique coding variants revealed a nonsense variant in the juvenile USA Brittany spaniel (stop gained, *DMD* p.Q2687*). Integrative Genomics Viewer (IGV) view provided in Figure 3.

**Figure 4:**
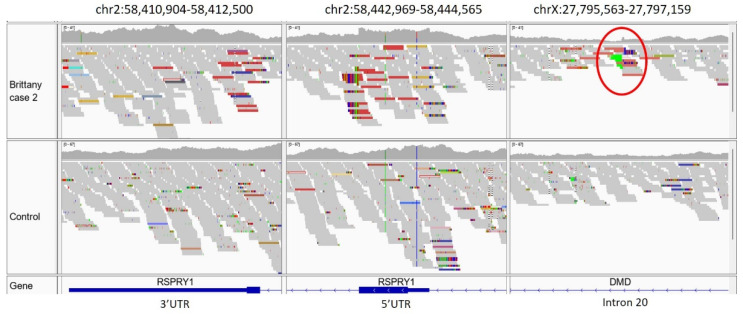
WGS reads from Brittany spaniel Case 2 aligned to dog chromosome 2 (**left** and **center**) and chromosome X (**right**) in the vicinities of the Ring Finger and *SPRY* Domain Containing 1 (*RSPRY1*) and *DMD* genes, respectively. The case is the top dog in all three panels. The colored bars circled in the case dog on the right represent reads that lie in intron 20 of *DMD* in which their mates map to the 3′ UTR or 5′ UTR (clusters of colored reads) of *RSPRY1*. WGS read mate pairs aligning to two different chromosomes can be indicative of a structural variant.

Case 2 (Japanese Brittany spaniel also reported in 2022 [30]). This dog was clear for the above stop gained p.Q2687* variant and was presumed to have a different *DMD* variant. No *DMD* coding variants were found in the VCF, but visual inspection of the WGS read alignments of the *DMD* gene revealed evidence for a retrogene insertion in intron 20 (Figure 4).

**Figure 5:**
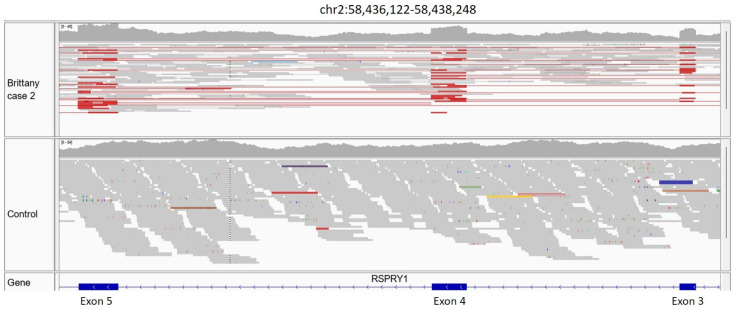
Evidence for the presence of a *RSPYR1* retrogene in Brittany spaniel Case 2. The red mate pairs aligned over consecutive exons of *RSPYR1* (skipping over intronic sequence but connecting red lines indicating their mates) are expected from reads derived from a retrogene-mediated *RSPYR1* cDNA insertion elsewhere in the genome. A similar result is not observed in the control dog.

Sanger sequencing of a PCR amplicon derived from a forward primer from *DMD* intron 20 and a reverse primer from *RSPRY1* exon 3 identified a contiguous exonic sequence from the 5′ UTR of *RSPYR1* to exon 3 (Sup Figure 1). In addition, sequencing of a PCR amplicon derived from a forward primer from *RSPYR1* 3′ UTR sequence and a reverse primer from *DMD* intron 20 revealed *RSPYR1* 3‴-UTR sequence and a poly A tail. The presence of target site duplication (TSD) sequences in both amplicons confirms their origin as a retrogene insertion (Figure 5).

### 3.4. Novel DMD Variant Identified in a 3-Month-Old Male Intact French Bulldog

**Figure 6:**
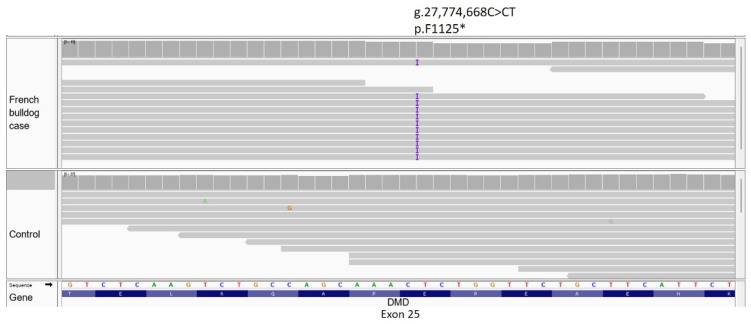
IGV view of a *DMD* 1 bp insertion mutation in exon 25 of a French bulldog with dystrophin-deficient MD. The purple “I” identified in all but one aligned read in the case is the insertion of a T at genomic DNA position 27,774,668, resulting in a p.F1125* mutation in the DMD protein.

Examination of this VCF for unique coding variants revealed a 1 bp insertion frameshift variant in *DMD*, resulting in a premature stop codon (*DMD*, p.F1125*, IGV presented in Figure 6).

## 4. Discussion

In this report, we updated all previously reported *DMD* variants along with their complete descriptions to CanFam4 (Table 1) and added our newly identified *DMD* variants in Brittany spaniels and the French bulldog. Table 1 also contains the current list of all other known mutations resulting in other forms of canine MD. Thus, in addition to the many known forms of dystrophin-deficient MD across many breeds, the spectrum of known canine MDs also includes forms of limb–girdle MDs in the Boston terrier [22] and miniature dachshund [23], and congenital MDs associated with variants in *COL6* in Landseer Newfoundland dogs [24], Labrador retrievers [25], and American Staffordshire terriers [26], in *LAMA2* in the Italian greyhound [27] and Staffordshire bull terrier [28], and in *LARGE1* in Labrador retrievers [29]. This list will no doubt continue to grow as muscle biopsies are pathologically characterized and new variants identified and correlated with the clinical presentations.

The original clinical report of dystrophin-deficient MD in Brittany spaniels [30] did not attempt to identify a causative mutation. In this report, we analyzed WGS from the Brittany spaniel originating in the United States (Case 1) and identified a nonsense mutation in *DMD* exon 55 resulting in a premature stop codon. Since the original case report, an additional 6-month-old male Brittany spaniel related to Case 1 was evaluated for progressive exercise intolerance, dysphagia first noted when the dog was 8 weeks of age, and muscle atrophy. A congenital muscle disease was suspected, muscle biopsies evaluated, and genotyping performed for the variant identified in Case 1. This second dog had the same stop gained DMD p.Q2687* variant as Case 1. Genetic testing of Brittany spaniels used for breeding should help eliminate this variant.

The Japanese Brittany spaniel (Case 2) in our original report [30] was clear for the variant affecting Case 1. However, further inspection of the WGS read alignments within the *DMD* gene was able to identify evidence of a retrogenic insertion of *RSPYRI* cDNA within intron 20. We suspect that the *RSPYR1* retrogene insertion within the *DMD* gene of Brittany spaniel case 2 is capable of disrupting *DMD* gene splicing and expression due to incorporation of the retrogene sequence into the initial transcript [36].

The discovery of a 1 bp insertion frameshift variant in *DMD* resulting in a premature stop codon in a young male French bulldog was also provided in this report. This novel variant was not found in any of the 22 other French bulldogs used in creating the VCF file. Although we cannot comment on the possibility of its presence in the dog population at large, it is possible that, like many/most *DMD* mutations discovered thus far, it is a variant isolated to this case. However, in 2009, an abstract was presented describing an 8-month-old male French bulldog in Brazil with clinical signs of dysphagia, dyspnea and weakness, with markedly elevated CK activity. Immunohistochemical staining and immunoblotting confirmed dystrophin deficiency [37]. Further, in 2012, a 5-month-old male French bulldog was reported with a 3-month history of dysphagia, regurgitation, exercise intolerance and delayed growth, and markedly elevated CK activity. Pathological diagnosis was of a dystrophic phenotype, and immunofluorescence staining and immunoblotting confirmed dystrophin deficiency [38]. At the time of those reports, WGS was not available for evaluation of *DMD* variants in these dogs. Recently, archived frozen muscle from the dog of the 2012 report was located, DNA extracted, and tested for the *DMD* genotype in the French bulldog reported here. That dog did not have the same 1 bp insertion frameshift variant as the French bulldog in this report. It is possible that other variants occur in French bulldogs, as more than one *DMD* variant has been identified in dystrophic Cavalier King Charles spaniels [12,16] and in Labrador retrievers [9,10,20,21]. Since French bulldogs have now become the #1 breed registered by the American Kennel Club (https://www.akc.org/expert-advice/dog-breeds/most-popular-dog-breeds (accessed on 1 July 2023)). Identification of muscle diseases and development of genetic testing to eliminate at-risk breeding animals is crucial.

## 5. Conclusions

The list of various forms of canine MD and affected breeds is certain to grow in the coming years with the advances in gene sequencing at affordable prices. Most canine inherited muscle diseases with variants identified to date have been associated with single genes and amenable to reduction or elimination by altered breeding practices. Complete clinical evaluations, pathological characterization of muscle biopsies and continued collaboration with geneticists are essential moving forward.

## Figures and Tables

**Table 1:** Classification of Muscular Dystrophies by Variant Identification.

Dystrophin Deficient Muscular Dystrophy							
Gene	Breed	Inherit.	Chr.	Genomic (g.)	Protein (p.)	Variant Phenotype Variant Phenotype	References
*DMD*	Golden Retriever	X-linked	X	g.28,126,496T>C	p.P178*	splicing, out-of-frame (exon 7 skipping)	Sharp et al., 1991, [6]
*DMD*	Rottweiler	X-linked	X	not reported nonsense mutation in exon 58	not reported	nonsense/stop-gain (exon 58)	Winand et al., 1994, [7]
*DMD*	German Shorthaired Pointer	X-linked	X			deletion encompassing the entire DMD gene	Schatzberg et al., 1999, [8]
*DMD*	Labrador Retriever	X-linked	X	not reported 184 nucleotide [pseudoexon] insertion between exon 19 and exon 20	p.E795*	pseudoexon/stop-gain (intron 19)	Smith et al., 2007, [9]; Kornegay et al., 2012, [10]
*DMD*	Jack Russell Terrier	X-linked	X	g.27,787,968_28,181,867del	p.G33*	deletion, out-of-frame (exons 3–21)	Brunetti et al., 2010, [11]
*DMD*	Cavalier King Charles Spaniel	X-linked	X	g.27,122,234G>A	p.R2396*	splicing, out-of-frame (exon 50 skipping) (exon 50 skipping)	Walmsley et al., 2010, [12]
*DMD*	Pembroke Welsh Corgi	X-linked	X	g.27,894,093_27,894,094insN[(480)]	p.V536*	pseudoexon/stop-gain (intron 13)	Smith et al., 2011, [13]
*DMD*	Cocker Spaniel	X-linked	X	not reported 4 bp deletion in exon 65	not reported	frameshift/stop-gain (exon 65)	Kornegay et al., 2012, [10]
*DMD*	Tibetan Terrier	X-linked	X	not reported large deletion of exons 8–29	p.D217*	deletion/stop-gain (exons 8–29)	Kornegay et al., 2012, [10]
*DMD*	Norfolk Terrier	X-linked	X	g.27,778,709del	p.G1029*	frameshift/stop-gain (exon 23)	Jenkins et al., 2015, [14]
*DMD*	Japanese Spitz	X-linked	X	g.27804717_33,211,801inv	p.E795*	~5.4 Mb inversion (intron 19)	Antencia-Fernandez et al., 2015, [15]
*DMD*	Cavalier King Charles Spaniel	X-linked	X	g.27,615,610_27,615,616del	p.N2021*	frameshift/stop-gain (exon 42)	Nghiem et al., 2016, [16]
*DMD*	Miniature Poodle	X-linked	X			>5 Mb deletion encompassing the entire DMD gene	Sanchez et al., 2018, [17]
*DMD*	Australian Labradoodle	X-linked	X	g.27,794,550G>A	p.R890*	nonsense/stop-gain (exon 21)	Shrader et al., 2018, [18]
*DMD*	Border Collie	X-linked	X	g.27,799,217del	p.N852*	frameshift/stop-gain (exon 20)	Lopez et al., 2018, [19]
*DMD*	Labrador Retriever	X-linked	X	g.27,795,539_30,022,040inv	p.D876*	~2.2 Mb inversion (intron 20)	Barthelemy et al., 2020, [20]
*DMD*	Labrador Retriever	X-linked	X	g.28,052,927_28,453,564dup	p.E12_D217dup	~400 kb duplication, in-frame (exons 2–7)	Shelton et al., 2022, [21]
*DMD*	Brittany Spaniel	X-linked	X	g.26,939,052G>A	p.Q2687*	nonsense/stop-gain (exon 55)	Current paper
*DMD*	Brittany Spaniel	X-linked	X	g.27,796,380ins *(RSPRY1* retrogene)	unknown	Retrogene insertion (intron 20)	Current paper
*DMD*	French Bulldog	X-linked	X	g.27,774,668C>CT	p.F1125*	frameshift/stop-gain (exon 25)	Current paper
**Limb Girdle Muscular Dystrophy**							
*SGCD*	Boston terrier	AR	4	g.54,241,112_54,241,113del	p.E178*	frameshift/stop-gain (exon 7)	Cox et al., 2017, [22]
*SGCD*	Boston terrier	AR	4	g.54,148,966-54,148,968delinsCC;54,148,978_54,168,381del]	p.R191*	~19.4 kb deletion (exons 8–9)	Cox et al., 2017, [22]
*SGCA*	Miniature Dachshund	AR	9	g.26,117,203G>A	p.W75*	nonsense/stop-gain (exon 3)	Mickelson et al., 2021, [23]
**Congenital Muscular Dystrophy**							
*COL6A1*	Landseer Newfoundland	AR	31	g.39,284,371G>T	p.E97*	nonsense/stop-gain (exon 3)	Steffen et al., 2015, [24]
*COL6A3*	Labrador retriever	AR	25	g.48,296,579G>A	p.R1576*	nonsense/stop-gain (exon 11)	Bolduc et al., 2020, [25]
*COL6A3*	Labrador retriever	AD	25	g.48,289,626C>T	p.G2053_P2070del	splicing (exon 17 skipping)	Bolduc et al., 2020, [25]
*COL6A3*	American Staffordshire Terrier	AR	25	g.48,287,602del	p.P2133*	frameshift/stop-gain (exon 21)	Jankelunas et al., 2023, [26]
*LAMA2*	Italian Greyhound	AR	1	g.68,441,978G>A	p.W1046*	nonsense/stop-gain (exon 22)	Christen et al., 2021, [27]
*LAMA2*	Staffordshire Bull Terrier	AR	1	g.68,292,960_68,295,150	p.I211_R270del	~2.2 kb deletion, in-frame (exon 5)	Shelton et al., 2021, [28]
*LARGE1*	Labrador retriever	AR	10	g.31,373,423C>T	p.R455*	nonsense/stop-gain (exon 11)	Shelton et al., 2021, [29]

All positions reported in UU_Cfam_GSD_1.0/canFam4. DMD: NM_001003343; COL6A1: XM_038581692.1; COL6A3: NM_001103215.1; LAMA2: XM_038526759.1; LARGE1: XM_038550745.1; SGCD: XM_038534931.1; SGCA: XM_038547640.1.

## Data Availability

Raw sequence reads are available in NCBI’s Short Read Archive at BioProject PRJNA982829.

## Supplementary Materials

The following supporting information can be downloaded at: https://www.mdpi.com/article/10.3390/genes14081557/s1, Figure S1: Sanger sequencing results for PCR amplicons designed to detect the retrogene-mediated insertion of a *RSPRY1* cDNA within intron 20 of the *DMD* gene. Table S1: A list of the unique coding variants of each dog.
