# Supplementary figures and images for: Current Classification of Canine Muscular Dystrophies and Identification of New Variants
Source: Genes (Basel). 2023 Jul 29;14(8):1557. doi: 10.3390/genes14081557 (PMC10454810; doi:10.3390/genes14081557)

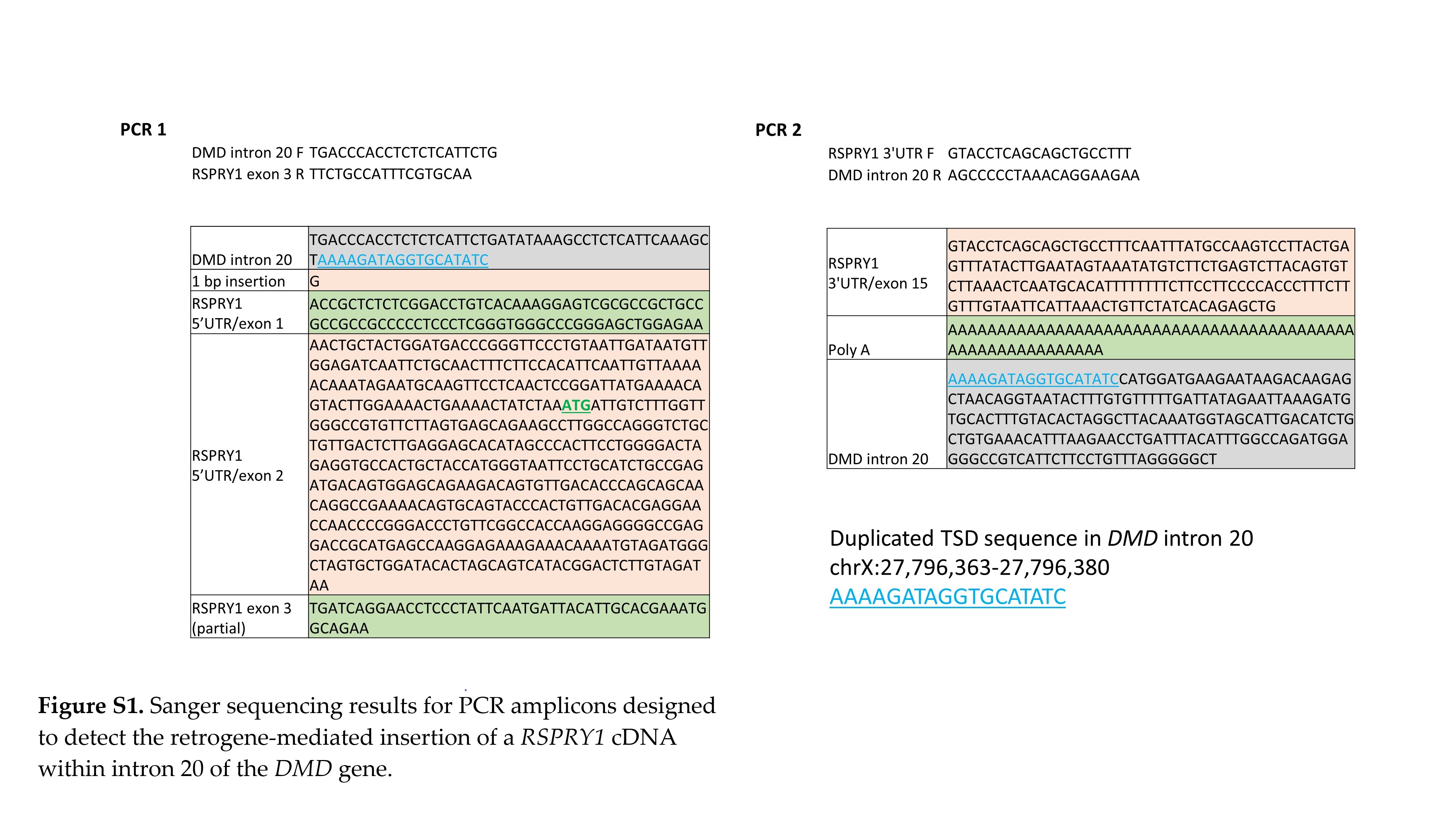

Supplement: Supplementary file 1 [file genes-14-01557-s001.zip › Figure S1.jpg]
